# *Candida*–streptococcal interactions in biofilm-associated oral diseases

**DOI:** 10.1371/journal.ppat.1007342

**Published:** 2018-12-13

**Authors:** Hyun Koo, David R. Andes, Damian J. Krysan

**Affiliations:** 1 Biofilm Research Laboratory, Department of Orthodontics, Divisions of Pediatric Dentistry and Community Oral Health, School of Dental Medicine, University of Pennsylvania, Philadelphia, Pennsylvania, United States of America; 2 Department of Medicine, Section of Infectious Diseases, University of Wisconsin–Madison, Madison, Wisconsin, United States of America; 3 Department of Medical Microbiology and Immunology, University of Wisconsin–Madison, Madison, Wisconsin, United States of America; 4 Department of Pediatrics, Carver College of Medicine, University of Iowa, Iowa City, Iowa, United States of America; 5 Department of Microbiology and Immunology, Carver College of Medicine, University of Iowa, Iowa City, Iowa, United States of America; Geisel School of Medicine at Dartmouth, UNITED STATES

## Bacterial–fungal interactions and oral diseases

The oral cavity contains up to 700 different species of microorganisms, including both bacteria and fungi [1]. The interactions of these communities of different organisms has become of increasing interest, particularly with respect to cross-kingdom interactions involving fungi and bacteria, which have been associated with severity of dental caries (tooth decay) and mucosal infections. Here, we provide a short review of the significance and mechanisms for the interactions between *C*. *albicans* and streptococci, the most common fungal and bacterial organisms in the oral cavity [2–5].

**Fig 1:**
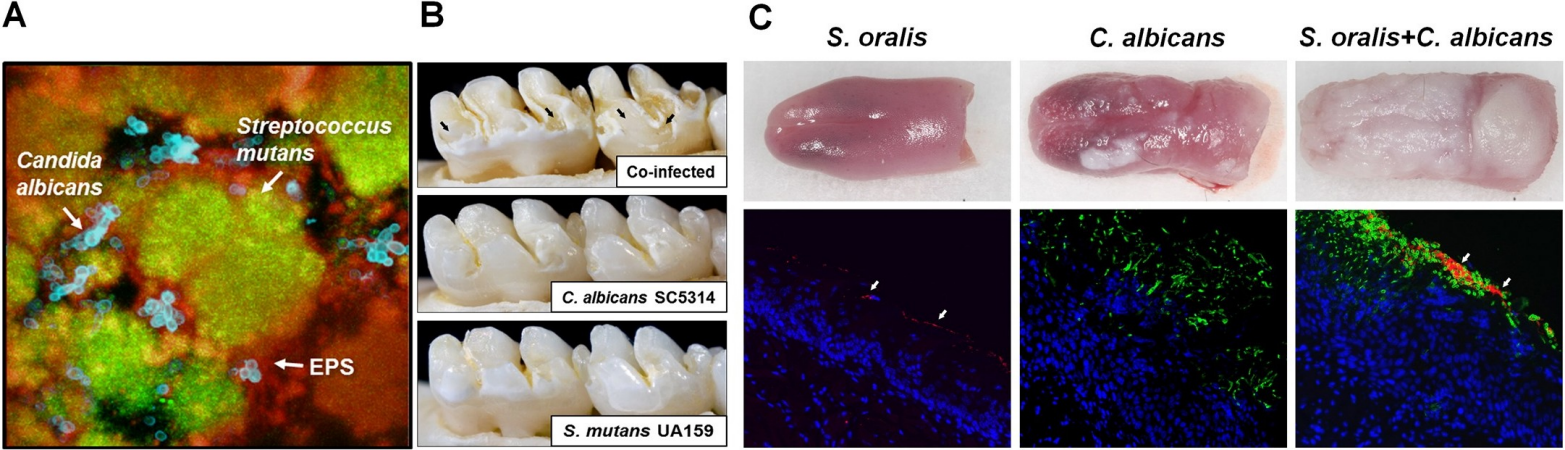
*Candida*–streptococcal interactions and oral diseases. A. Confocal fluorescence microscopy images of *C*. *albicans*–*S*. *mutans* mixed biofilms, illustrating the spatial relationship between *C*. *albicans* (blue), *S*. *mutans* (green), and exopolysaccharides (red). B. Images of teeth from rats infected with *S*. *mutans*, *C*. *albicans*, or coinfected. Black arrows indicate severe carious lesions of coinfections in which enamel is missing, which exposes underlying dentin. Such rampant caries was absent in the animals infected by *S*. *mutans* or *C*. *albicans* alone. C. Fluorescence microscopy images of harvested mouse tongues infected with *S*. *oralis* (red, see arrows), *C*. *albicans* (green), or both. Coinfection substantially increased bacterial–fungal biofilm accumulation, soft tissue invasion, and inflammatory response. *Original images provided by Dr*. *Anna Dongari-Bagtzoglou; adapted from Sobue T*. *and colleagues*, Methods Mol Biol. *1356*:*137–52*, *2016*, *with permission*. EPS, exopolysaccharides.

*Candida albicans* and oral streptococci coinfections are associated with enhanced virulence of dental caries and more severe oropharyngeal diseases (Fig 1) [6,7]. Specifically, *C*. *albicans* partners with *Streptococcus gordonii*, *S*. *oralis*, and *S*. *sanguinis* to enhance bacterial colonization and biofilm formation. In addition, *C*. *albicans* becomes more invasive, exacerbating mucosal tissue infection and destruction [8,9]. Mixed *C*. *albicans–*bacterial infections are also associated with denture stomatitis, the inflammation of the oral mucosa under dentures. Furthermore, *C*. *albicans*–bacterial communities have been clinically found in other oral niches, including periodontal pockets and endodontic canals [6].

*C*. *albicans*–streptococcal biofilms are an important contributor to the development of early childhood caries that affects toddler-age children [10–12]. Severe childhood caries is a particularly virulent form of caries that causes extensive and painful tooth destruction, induced by protracted consumption of sucrose containing foods and beverages [11]. Typically, *C*. *albicans* is usually absent on teeth of healthy, caries-free children [10]. Furthermore, *C*. *albicans* does not interact strongly with *S*. *mutans* (a caries-causing pathogen), nor is it an efficient colonizer of mineralized tooth enamel by itself. However, the high level of sucrose in the oral cavity increases the physical coadhesion between the *C*. *albicans* and *S*. *mutans* as well as tooth surface colonization and drastically enhances the microbial burden, aciduricity, and production of extracellular matrix. Ultimately, the extensive mixed-kingdom and acidogenic biofilm leads to severe tooth decay in a process that can be recapitulated in a rodent model under sugar-rich diets [12].

## Fungal and bacterial cell surface adhesins mediate C. *albicans* interactions with mitis group streptococci on mucosal surfaces

**Fig 2:**
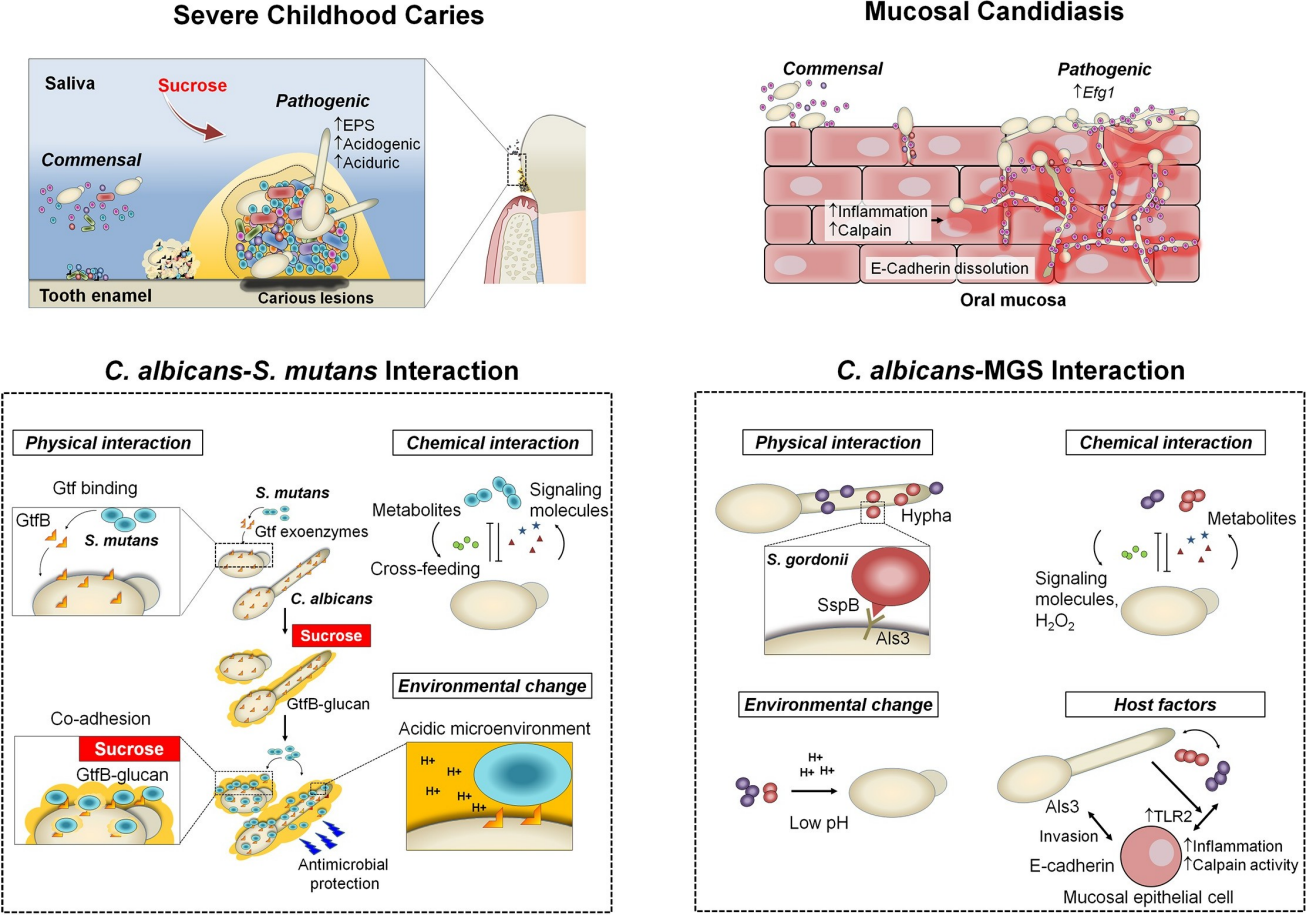
Pathogenic mechanisms of *C*. *albicans*–oral streptococcal cross-kingdom interactions. Complex physical and chemical interactions (including cross-feeding and metabolites exchange) as well as environmental and host factors govern the development of pathogenic bacterial–fungal biofilms, including spatial organization, virulence, and drug protection/resistance. These interactions can be cooperative or competitive to mediate symbiotic, antagonistic, or synergistic relationships, often modulated by host and environmental factors to promote the onset and amplify the severity of the disease. Host diet (dietary sugars, particularly sucrose) promote the interactions between *C*. *albicans* and *S*. *mutans* by providing a substrate for EPS α-glucans production by streptococcal Gtfs that enhances coadhesion and bacterial–fungal tooth colonization, stimulating cross-kingdom biofilms. This interaction enhances the carriage of the cariogenic pathogen and acid production, while the presence of *C*. *albicans* increases EPS matrix production (via Gtf induction and fungal-derived EPS) and biofilm aciduricity, resulting in cariogenic conditions on tooth surface. Likewise, the pathogenic impact of *C*. *albicans* interactions with MGS on mucosal surfaces is also influenced by host factors. The interactions of *S*. *oralis* with *C*. *albicans* on mucosal surfaces cause exacerbated inflammatory responses and increased neutrophilic activity. *C*. *albicans* increase the biomass of *S*. *oralis* and this leads to increased mucosal TLR2 expression, activating proinflammatory signaling. *C*. *albicans* and *S*. *oralis* also synergistically increase epithelial μ-calpain activity, a proteolytic enzyme that targets E-cadherin from epithelial junctions. The bacteria influence fungal physiology by promoting hyphal formation via the Efg1 filamentation pathway and expression of secreted aspartyl proteases, which further induces proteolytic degradation of E-cadherin, facilitating invasion and tissue destruction. Efg1; EPS, exopolysaccharides; Gtf, glucosyltransferase; MGS, mitis group streptococci; TLR2.

*C*. *albicans* physically interacts with mitis group streptococci (MGS) species such as *S*. *gordonii*, *S*. *sanguinis*, and *S*. *oralis* through well-characterized cell wall surface proteins/receptors on both organisms [6,13]. Streptococcal cell surface adhesins SspA and SspB (from the antigen I/II polypeptide family) interact with the *C*. *albicans* surface, while *ALS* and *HWP1* adhesins on the fungal cell wall appear to mediate binding to MGS (Fig 2). Specifically, SspB and Als3 directly bind *C*. *albicans* and *S*. *gordonii* together through the N-terminal domain of Als3 [14]. These interactions may also involve *O*-mannosyl residues in Als adhesins and other cell wall proteins, such as Sap9 [15,16].

The consequences of *C*. *albicans*–streptococcal interactions have been demonstrated in vivo. *C*. *albicans* and *S*. *oralis* coinfection results in increased tissue invasion and heightened mucosal inflammatory responses compared with infection by either organism alone [9]. This latter feature appears to be due to increased induction of multiple neutrophil-activating cytokines and up-regulation of TLR2-dependent inflammatory genes as well as enhanced epithelial μ-calpain activity [9,17] (Fig 2).

## *C*. *albicans* interacts with *S*. *mutans* exoenzymes (glucosyltransferases) to promote interspecies biofilm formation on tooth surface

In contrast to MGS, *C*. *albicans* does not directly bind to the cariogenic pathogen *S*. *mutans*. Instead, glucosyltransferases (Gtfs) secreted by *S*. *mutans* promote the generation of an extensive extracellular matrix in the presence of *C*. *albicans*, leading to virulent mixed biofilms under sugar-rich conditions of severe childhood caries [18,19]. *S*. *mutans*-derived GtfB binds avidly to the *C*. *albicans* cell surface and converts sucrose to large amounts of extracellular polysaccharides (EPS) α-glucans on the fungal surface (Fig 2). The EPS provides bacterial binding sites for *S*. *mutans* and concurrently allows *C*. *albicans* to bind to and colonize teeth [12]. Consequently, the interaction between *S*. *mutans* and *C*. *albicans* is mediated by both secreted Gtfs and their glucan product. This mechanism is distinct from the more typical cell–cell binding interactions observed between MGS, staphylococci, or bacillus and *C*. *albicans*, although the role of Gtfs in MGS species has not been extensively studied [13].

To further understand the mechanistic basis of this “biochemical interaction,” we identified the *C*. *albicans* surface molecules to which GtfB binds. *C*. *albicans* mutants lacking either *N*- or *O*-linked mannans (located on the outer most layer of the fungal cell wall) showed severely reduced GtfB binding. As a result, these mannoprotein-deficient mutants developed poor mixed-species biofilms with *S*. *mutans*, showed reduced EPS α-glucans content, and reduced microbial carriage on teeth in vivo [18]. Likewise, *S*. *mutans* defective in GtfB does not yield mixed-species biofilms with *C*. *albicans*.

In a rodent model, the sucrose-dependent partnership between *C*. *albicans* and *S*. *mutans* synergistically enhanced bacterial–fungal carriage within plaque biofilm, leading to aggressive onset of tooth decay with rampant carious lesions similar to those found in severe childhood caries [12]. The potential mechanisms for severe caries have been an active subject of research, which entails at least, in part, enhanced microbial carriage, cross-feeding metabolic interactions, and the accumulation of adherent acidic biofilms on teeth facilitated by an EPS matrix surrounding acidogenic–aciduric organisms, as reviewed recently [5,10] (Fig 2).

## The role of the *C*. *albicans* master regulator Efg1 is required for MGS mixed biofilms but not for *S*. *mutans* mixed biofilms

*C*. *albicans* Efg1 regulates key hyphae-associated biofilm effector molecules, and homozygous efg1 deletion mutants form only rudimentary biofilms [20]. The nature of the biofilm formed between *S*. *mutans* and *C*. *albicans* differs from single species *C*. *albicans* biofilms because deletion of two transcription factors that are essential for *C*. *albicans* biofilms, Efg1 and Bcr1, does not affect the amount of fungal cells in the mixed biofilm. This is likely due to the fact that GtfB binds these mutants with similar afinity compared to wild types and generates robust extracellular α-glucans matrix that allows *C*. *albicans* to coadhere and form biofilm with *S*. *mutans* [18].

In contrast, *efg1*ΔΔ mutants are unable to form mixed biofilms with *S*. *oralis* [21]. Interestingly, overexpression of the Efg1-regulated adhesin *ALS1* partially restores *C*. *albicans*–*S*. *oralis* biofilm formation to *efg1*ΔΔ, suggesting that Als1 is a key mediator of this mixed biofilm [21]. Consistent with this notion, *C*. *albicans* strains lacking either *ALS1* or *ALS3* also are deficient for *S*. *oralis* mixed biofilm formation [21]. Als3 is also crucial for *C*. *albicans*–*S*. *gordonii* mixed biofilms through a mechanism involving an interaction between Als3 and SspB [22]. However, *C*. *albicans* strains lacking *ALS3* are able to form mixed biofilms with *S*. *mutans* under sucrose-rich conditions, showing similar levels of fungal cells as those formed with wild-type strains [18]. Thus, the interactions of *C*. *albicans* with oral streptococci vary significantly with the specific species of bacteria. Additional studies will be needed to understand how these differences affect the colonization and disease severity at distinct oral niches.

## *C*. *albicans*–bacterial biofilm relationship is critically dependent on EPS matrix and chemical interactions

The EPS matrix critically influences the relationship between *C*. *albicans* and oral streptococci within the biofilm [23]. The matrix provides a scaffold for both surface adhesion and cell-to-cell cohesion while at the same time establishing chemical and nutrient gradients by modulating diffusion [5]. Like most microbes, the matrix of *Candida* species is comprised of the protein, carbohydrate, nucleic acid, and lipids. In particular, a complex containing mannan and β-glucan constituents sequesters antifungal drugs to protect *Candida* cells from their effects [23]. Nearly a dozen *C*. *albicans* proteins involved in polysaccharide synthesis and modification (e.g., Phr1, Bgl2, Alg11, and Mnn11) are indispensable for production of the matrix [23]. In mixed biofilms, the fungal derived biofilm matrix also protects some prokaryotic pathogens (e.g., *Staphylococcus aureus* and *Escherichia coli)* against antibacterial drugs [24]. Similarly, *S*. *mutans*-derived α-glucans surrounding fungal cells form an additional “drug-trapping matrix” that prevents uptake of the antifungal fluconazole, reducing killing efficacy [25].

Complex signaling, cross-feeding, and metabolic interactions within the biofilm shape its microenvironment and lead to pathogenic synergies that modulate the onset and severity of oral diseases (Fig 2). A range of signaling/quorum sensing (QS) molecules and other factors appear to facilitate these synergies, including AI-2, peptidoglycan fragments, exoenzymes, and hydrogen peroxide (H_2_O_2_) [2–4,13,26]. For example, nutrient byproducts as well as AI-2 signaling and H_2_O_2_ from *S*. *gordonii* stimulate *C*. *albicans* hyphal development within the biofilm [26], while *S*. *oralis* presence also activates expression of fungal aspartyl proteases [13]. Conversely, *C*. *albicans* can promote streptococcal proliferation by providing growth-stimulating factors and reducing oxygen tension [13,26]. The impact of *C*. *albicans* and MGS synergism on the host–pathogen interaction has been demonstrated in vivo whereby mixed biofilm (with *S*. *oralis*) growth enhances neutrophil infiltration, leading to increased severity of soft tissue lesions [9,17] (Fig 2). This is distinct from single-species *C*. *albicans* biofilms, which are notable for inhibition of neutrophil influx and subsequent function.

A further example of the consequences of the EPS matrix and chemical interactions has been observed between *C*. *albicans* and *S*. *mutans*. *S*. *mutans* converts sucrose to glucose that can be more readily metabolized by *C*. *albicans* [27,28, 29]. Importantly, *C*. *albicans* activates *S*. *mutans* competence [28], virulence genes, and GtfB production via QS molecules such as farnesol [27]. Furthermore, *C*. *albicans* secretes its own matrix products such as β-glucan and creates an EPS-producing loop within the *S*. *mutans* mixed biofilm [12]. As a result, the organisms enhance the carriage of cariogenic pathogens, biofilm accumulation, and acid production, promoting a localized and persistent acidogenic–aciduric microenvironment that potentiates demineralization of tooth enamel and may explain the synergistic enhancement of caries severity.

Although the cross-kingdom synergies are involved in the pathogenesis of both mucosal and dental diseases, the interactions can also repress functions of the member species to modulate population growth, biofilm structure, community changes, and spatial organization [5] (Fig 2). For example, *S*. *mutans*-derived metabolites such as mutanobactin A and fatty acid signaling trans-2-decenoic acid inhibit *C*. *albicans* hyphal formation [30,31]. These effects, in addition to the generation of a hyphae-inhibiting, acidic environment, can explain why yeast forms are associated with *S*. *mutans* clusters in the deeper layers of mixed biofilms [12]. Furthermore, competence-stimulating peptides released by *S*. *mutans* [32] and *S*. *gordonii* [33] also disrupt hyphal formation in *C*. *albicans* cells. These hyphae-inhibiting effects are consistent with the fact that the Efg1 filamentation pathway is not required for mixed *C*. *albicans*–*S*. *mutans* biofilm growth and cariogenicity as noted above, suggesting that in contrast to mucosal candidiasis, filamentation may not be a virulence-promoting phenotype in mineralized tissue infections such as dental caries. Paradoxically, farnesol produced by *C*. *albicans*, which stimulates *S*. *mutans* growth and *gtfB* expression at low concentrations (25–50 μM), disrupts bacterial growth at high concentrations (>100 μM) [27]. Therefore, a tightly regulated cooperative and antagonistic balance through stimulus-inhibition mechanisms appears to mediate bacterial–fungal coexistence and survival within biofilms, which can become synergistic when conditions are conducive for disease (Fig 2).

In summary, the polymicrobial nature of biofilm-associated oral diseases has been increasingly recognized. Clinical data, together with in vivo studies, provide compelling evidence of the importance of cross-kingdom interactions in the severity of mucosal diseases and dental caries. Complex cell–cell and cell–EPS matrix interactions, spatial organization, and chemical/metabolic factors modulate biofilm development and virulence. These fungal–bacterial interactions are facilitated by host factors (immunity, diet, and salivary function) to modify the local microenvironment and promote oral diseases. Elucidating how bacterial–fungal interactions occur spatiotemporally (cooperative, competitive, or both simultaneously) to mediate symbiotic, antagonistic, or synergistic states may shed new light into the pathogenic mechanisms and identify more effective therapeutic targets. Since cross-kingdom biofilms exist throughout the gastrointestinal tract, principles and molecules that emerge from these studies may lead to novel approaches to prevent and eradicate other intractable polymicrobial biofilms at various clinical niches.
